# Determinants of Sexual Dysfunction in Parkinson’s Disease Patients: A Secondary Analysis of a Multicenter Cross-Sectional Study

**DOI:** 10.3390/jcm14010152

**Published:** 2024-12-30

**Authors:** Alfredo Manuli, Maria Grazia Maggio, Paolo De Pasquale, Loredana Raciti, Serena Filoni, Simona Portaro, Gianluca Pucciarelli, Rocco Salvatore Calabrò

**Affiliations:** 1 Department of Biomedicine and Prevention, University of Rome Tor Vergata, 00133 Rome, Italy; manulialfredo@gmail.com (A.M.); g.pucciarelli81@gmail.com (G.P.); 2 IRCCS Centro Neurolesi Bonino Pulejo, 98124 Messina, Italy; paolo.depasquale@irccsme.it (P.D.P.); salbro77@tiscali.it (R.S.C.); 3 Unità Spinale Unipolare, AO Cannizzaro, 95126 Catania, Italy; loredana.raciti79@gmail.com; 4 Unit of Neuro-Rehabilitation, IRCCS “Casa Sollievo della Sofferenza”, 71013 San Giovanni Rotondo, Italy; serena.diba@gmail.com; 5 A.O.U. Policlinico “G. Martino”, Via Consolare Valeria, 98124 Messina, Italy; simonaportaro@hotmail.it

**Keywords:** non-motor symptoms, neurorehabilitation, sexual health, neurodegenerative disorders, gender

## Abstract

**Background**: Sexual dysfunction (SD) in Parkinson’s Disease (PD) patients is a common and distressing concern, although it remains an underdiagnosed and undertreated condition. Indeed, the prevalence of SD in PD ranges from 42.6% to 79% in men and from 36% to 87.5% in women. PD-related SD etiology is multifactorial and includes biological, psychological, and socio-relational factors. In a previous multicenter study on 203 PD patients, we found that there was no gender difference in dysfunction severity, although men were significantly more affected by SD than women. **Methods**: This paper is a secondary analysis of this previous multicenter study, and aims to investigate the potential risk factors that contribute to SD. The relationship between dysfunction and the experimental factors was assessed using Generalized Linear Mixed-Effects (GLME) model. **Results**: The final analysis was conducted on a sample of 177 patients (76 women), after excluding individuals with incomplete data, non-compliance with inclusion criteria, or delays in assessment tool administration. The analysis, performed using the GLME model (R^2^ = 0.68), revealed that gender (*p* = 0.01), age (*p* = 0.02), and depression status (HAMD) (*p* = 0.01) were significant predictors of SD. In contrast, other factors considered in the model, such as diabetes, and autonomic symptoms, did not significantly influence SD. **Conclusions**: This study demonstrates that age, gender, and depression are important predictors of SD in patients with PD. Although SD is a common NMS in PD patients, it is often neglected in clinical practice and the research on this hot topic is still poor. Then, a multidisciplinary approach, including nursing and coaching, is mandatory in order to improve sexual health in this patient population.

## 1. Introduction

Parkinson’s Disease (PD) is a neurodegenerative disorder primarily affecting the substantia nigra and leading to dopamine deficiency. This results in hallmark motor symptoms, such as resting tremor, rigidity, bradykinesia, and shuffling gait [1].

The diagnosis of PD is typically based on the Movement Disorder Society (MDS) clinical diagnostic criteria, which are widely regarded as the most current and widely used standards. These criteria require the presence of bradykinesia combined with either rigidity, rest tremor, or both, alongside the exclusion of alternative diagnoses as well as supportive criteria, such as responsiveness to dopaminergic therapy [2]. The UK Parkinson’s Disease Society Brain Bank clinical diagnostic criteria have also been widely utilized in the research and clinical practice, requiring the presence of bradykinesia with either rigidity or rest tremor, alongside exclusion criteria and supportive features to confirm the diagnosis [3].

PD is estimated to affect approximately 1–2% of individuals aged over 65 years worldwide [4], making PD the second most-common neurodegenerative disorder after Alzheimer’s Disease.

The research has recently focused on the growing importance of non-motor symptoms (NMSs), which can appear years before motor impairment, negatively affecting quality of life [5,6]. Among these NMSs, dysautonomia is a key feature, occurring in up to 70–80% of PD patients during the disease course [7]. Dysautonomia symptoms include orthostatic hypotension, urinary dysfunction, and constipation, the latter affecting up to 80% of PD patients [8].These autonomic disturbances significantly impact quality of life and overlap with PD-related disorders, such as atypical parkinsonism, reflecting shared pathophysiological mechanisms [9].

Sexual dysfunction (SD) is a distressing NMS that requires greater attention, as both patients and healthcare professionals often underestimate it [10,11]. SD in PD patients includes erectile dysfunction (ED), premature ejaculation, decreased libido, and orgasm disorders in men, while women typically experience a reduced libido and difficulty achieving an orgasm. The prevalence of SD in PD ranges from 42.6% to 79% in men and from 36% to 87.5% in women, although its etiology remains unclear [12,13,14,15]. The complexity of PD-related SD is further compounded by factors such as dopaminergic therapies, which can induce hypersexuality or worsen SD through dopamine dysregulation [11,12,13].

Despite the growing recognition of SD as a common NMS in PD patients, it remains an underdiagnosed and undertreated condition [13]. Several factors contribute to this underreported problem, including patient embarrassment, reluctance to discuss sexual health with healthcare professionals, and a lack of awareness among clinicians [11,12,14]. Moreover, commonly used clinical tools, such as the Unified Parkinson’s Disease Rating Scale (UPDRS) and the Parkinson’s Disease Questionnaire (PDQ-39), neither address sexual function nor delve into it beyond a superficial level [14]. The lack of standardized assessment tools for sexual function in PD patients has hampered systematic evaluations as well as the understanding of SD and its impact on quality of life [11,13,15]. Moreover, these tools do not pay attention to gender differences, even if the issue is of growing importance. In recent years, some studies have addressed this gap by investigating the difference in prevalence and severity of SD in men and women with PD [16]. While men generally report a higher prevalence of SD, particularly erectile dysfunction [13,17], the growing evidence suggests that women with PD may also experience significant sexual health problems [17,18]. Identifying the risk factors associated with SD in PD patients is essential for developing targeted interventions [11,17].

Our previous multicenter observational study [14] investigated the prevalence of SD and associated variables, including gender differences, in a sample of 203 PD patients (113 men and 90 women) from three Italian regions. We found SD to be present in 68% of men and 53% of women, with reduced libido being the most commonly reported issue in both sexes. Although men were significantly more affected by SD, there was no gender difference in dysfunction severity. These findings highlight the need to address sexual health in PD management, as SD significantly affects patients’ quality of life [14].

Given the significant impact of SD on the quality of life in individuals with PD and the limited understanding of its multifactorial etiology, this study aimed to explore the key factors influencing SD in this population. Specifically, we sought to investigate the roles of demographic variables (e.g., age and gender), psychological factors (e.g., depression severity), and clinical parameters in predicting SD. We hypothesized that some of these variables would emerge as significant predictors, reflecting their established relevance in PD patients. We employed a Generalized Linear Mixed-Effects model to quantify these relationships and provide a more nuanced understanding of the biological, psychological, and social contributors to SD in PD patients. The findings are expected to inform future multidisciplinary interventions targeting this often-overlooked non-motor symptom.

## 2. Materials and Methods

### 2.1. Study Design and Setting

This is a secondary analysis of the database from our previous multicenter cross-sectional study involving participants diagnosed with PD according to the UK Parkinson’s Disease Society Brain Bank clinical diagnostic criteria. The original study was designed to assess the prevalence of SD and associated factors in PD patients from three different regions in Italy: the IRCCS “Centro Neurolesi Bonino-Pulejo” (located in Sicily), the “Fondazione Centri di Riabilitazione Gli Angeli di Padre Pio” (located in Puglia), and the Ospedale Motiggia Pelascini (situated in Lombardy).

The previous multicenter study [14] was approved by the local Ethics Committee of the IRCCS Centro Neurolesi Bonino-Pulejo (IRCCSME-SEXPD-39-2018); for secondary and post hoc analyses, there was no need for further approval.

### 2.2. Study Population

This study initially included 203 patients (113 men and 90 women) aged 60–80 years, with Hoehn–Yahr stages ranging from 1 to 3. The subjects were recruited based on the criteria established by Raciti et al. [14]. These criteria required a confirmed diagnosis of Parkinson’s Disease (PD) according to the UK Parkinson’s Disease Society Brain Bank clinical diagnostic criteria [19], the absence of moderate to severe cognitive impairment (MMSE score < 16), and no severe medical or psychiatric comorbidities interfering with participation. Patients with significant sensory deficits that could hinder their ability to complete assessments were also excluded.

For the purpose of this secondary analysis, the sample was refined to 177 patients (76 women) due to several factors: (1) incomplete data, as a significant number of patients failed to complete all required questionnaires, compromising the reliability of the results; (2) non-compliance with inclusion criteria over time, as changes in health status or disease progression were observed in some patients; and (3) delays in administering assessment tools, which impacted the accuracy of the data collected regarding SD.

To ensure the rigor of this refinement process, we conducted a thorough assessment of the characteristics of the excluded patients, comparing them to those retained in the final sample. Demographic (e.g., age and sex) and clinical characteristics (e.g., Hoehn–Yahr stage and MMSE score) were analyzed to confirm that the exclusions did not introduce significant biases into the study population. Statistical comparisons revealed no significant differences between the original cohort and the refined sample, supporting the representativeness of the final dataset. This rigorous approach ensured that the exclusion of cases was methodologically sound and preserved the validity and robustness of subsequent analyses.

**Figure 1:**
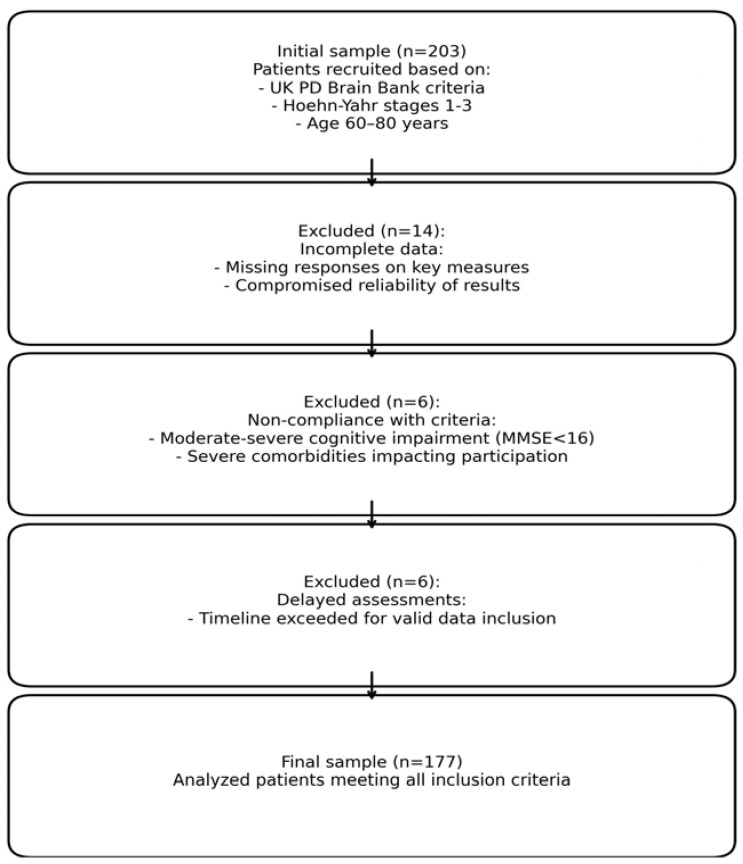
Flowchart of the selection processes.

To ensure clarity, we included a flow diagram (Figure 1) summarizing the inclusion and exclusion processes, detailing the adjustments made for this secondary analysis.

### 2.3. Outcome Measure

Sexual function was investigated using a semi-structured interview, the International Index of Erectile Function (IIEF), and the Female Sexual Function Index (FSFI). Each instrument was selected based on its relevance to the domains of SD explored in this study and its prior validation in clinical populations.

The semi-structured interview was a 40-item ad hoc tool developed by the authors to investigate three main areas of the patient’s life: sociodemographic information, illness perception, and sexuality. The data collected included sex, age, educational level, employment status, marital status, presence of son/daughter, disease burden, duration of illness, associated treatments, and the impact of the disease on sexuality and relationships. Additionally, the interview examined aspects of the patient’s past and present sexual life. This tool was specifically designed to address the gaps in standardized assessments and provide a more tailored understanding of the unique challenges faced by the patients in this study.

The IIEF-15 [20] is a standardized tool with 15 questions about the patients’ sexual experiences over the preceding 4 weeks, investigating five separate domains of sexual function: erectile function, orgasmic function, sexual desire, intercourse satisfaction, and overall satisfaction. A score less than or equal to 25 was considered indicative of ED. The IIEF is a widely validated tool in various clinical settings, including populations with chronic diseases, though its specific application to Parkinson’s Disease has been less extensively explored.

On the other hand, the FSFI [21] is a widely used measure of female SD, evaluating six domains: desire, arousal, lubrication, orgasm, satisfaction, and pain. The FSFI has demonstrated strong psychometric properties and is considered a gold standard for assessing female sexual health, including in populations with chronic conditions.

Clinical assessments included the UPDRS, which is the most commonly used tool to evaluate motor and non-motor symptoms in Parkinson’s Disease patients, and the Hamilton Depression Rating Scale (HAMD), employed to assess the emotional state of participants. Both scales are validated and extensively used in PD research.

As determinants of sexual dysfunction, we also considered demographic factors, such as age, gender, and education, due to their well-documented influence in the literature.

### 2.4. Statistical Analysis

The sample participants’ sociodemographic and clinical parameters were analyzed, and differences between male and female groups were assessed.

Descriptive statistics were analyzed and expressed as mean ± standard deviation for continuous variables; frequencies (%) were used for categorical variables. Age was treated as a continuous variable, while education and HAMD categories were analyzed as categorical variables. Similarly, the binary variables (i.e., dysfunction) were also considered categorical. For the continuous variable, normality was tested using the Shapiro–Wilk test (MATLAB function swtest). Since the age distribution for both the male and female groups was not normal, a nonparametric test, Mann–Whitney (MATLAB function ranksum) was applied to evaluate differences between genders. For the categorical variables (education, presence of son/daughter, HAMD, and dysfunction), a contingency table was constructed. Expected frequencies were calculated, and Fisher’s exact test (MATLAB function fishertest) was used when low frequencies were detected; otherwise, a chi-squared test (MATLAB function chi2cdf) was applied.

The relationship between dysfunction and the experimental factors was assessed using the Generalized Linear Mixed-Effects (GLME) model, implemented with the MATLAB function fitglme (MATLAB, 2022a, MathWorks, Natick, MA, USA). These models account for interindividual variability by including participants as a random effect. The initial set of fixed-effects candidate variables (gender, age, education, presence of a son/daughter, comorbidities, hypertension, cardiovascular disorders, diabetes, autonomic symptoms, emotional reactions, Levodopa drug, another drug—not for PD, UPDRS total, FSFI, IIEF-15, HAMD) was selected based on the previous literature and clinical relevance [22,23,24,25,26].

The fixed effects, including interactions, were selected using a stepwise procedure guided by the Akaike Information Criterion (AIC). The final model retained gender (G), age (A), diabetes (D), autonomic symptoms (As), HAMD score (H), and the interaction between As and H (AsH) as predictors of the response variable.

Categorical variables were coded as follows: G (0 = male, 1 = female), D (0 = no diabetes, 1 = diabetes), As (0 = none, 1 = yes, 2 = constipation), and H (0 = absent, 1 = mild, 2 = moderate, and 3 = severe depression). Other candidate variables were excluded based on inclusion criteria aimed at optimizing model fit and effectively explaining the response variable.

The model equation is represented as:

Y = g(u_0_ + α_0_ G + β_0_ A + λ_0_ D + ζ_0_ As + δ_0_ H + η_0_ AsH + ϵ) (1)

In this equation, g represents the link function, u_0_ is the individual intercept accounting for interindividual differences, and the coefficients (α_0_, β_0_, λ_0_, ζ_0_, δ_0_, η_0_) represent the fixed effects, reflecting the influence of G, A, D, As, H, and their interaction (AsH) on the response variable.

ϵ is the error term. Since the dysfunction data follow a binomial distribution (Y = 1 for dysfunction, Y = 0 for no dysfunction), a logit link function was applied to the GLME model. Model parameters were estimated using the maximum likelihood with Laplace approximation.

The significance of each fixed-effect term in the selected model was tested using the Wald Test [27], which evaluates the contribution of each fixed effect based on its coefficients and associated covariance structure.

## 3. Results

The final analysis was conducted on a sample of 177 patients (76 women), after excluding individuals with incomplete data, non-compliance with the inclusion criteria, or delays in the administration of assessment tools. This refined cohort ensures a more reliable evaluation of the factors influencing SD. For more details on the sample, see Table 1.

The analysis, performed using the GLME model (R^2^ = 0.68) and evaluated through the Wald Test, revealed that gender (*p* = 0.01), age (*p* = 0.02), and depression status (HAMD) (*p* = 0.01) were significant predictors of SD. In contrast, other factors included in the model, such as D (*p* = 0.19), As (*p* = 0.90) and the interaction AsH status (*p* = 0.07), did not show a significant effect on SD.

**Figure 2:**
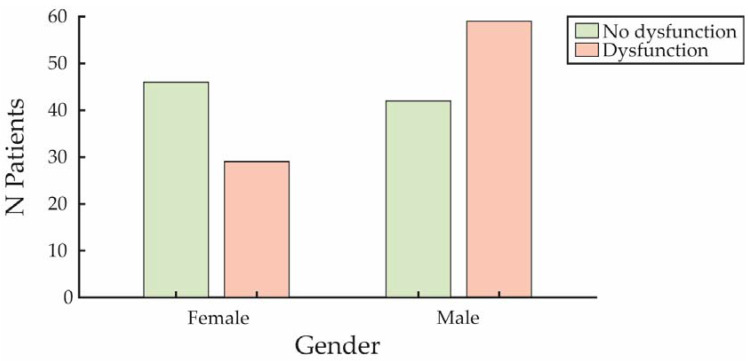
Distribution of SD by gender. The numbers of patients affected by sexual dysfunction (in red) and those unaffected (in green) are shown for both females and males.

Male participants showed a higher incidence of SD compared to females (Figure 2).

**Figure 3:**
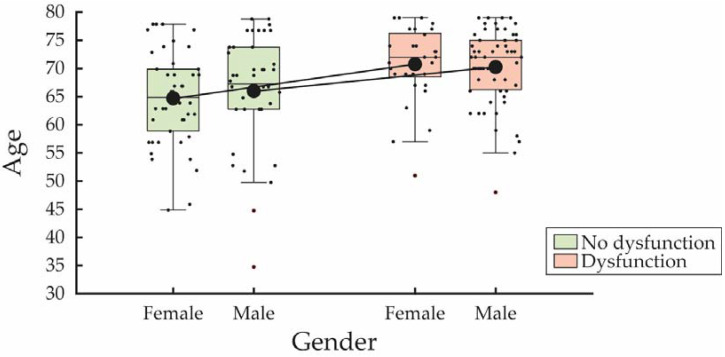
Sexual dysfunction as a function of age and gender. Box plots display the age distribution of patients with (in red) and without (in green) sexual dysfunction for both females and males. The solid lines connect the mean ages of the dysfunctional and non-dysfunctional groups for each gender.

Additionally, age significantly influenced SD in both genders, with a more pronounced effect observed in females. This is illustrated by the greater difference in mean ages between the dysfunctional and non-dysfunctional groups among women, as evidenced by the steeper slope of the connecting line (Figure 3).

Regarding depression (HAMD categories), the sample distribution was uneven, with few patients classified as not having depression (n = 8, of whom reported SD).

**Figure 4:**
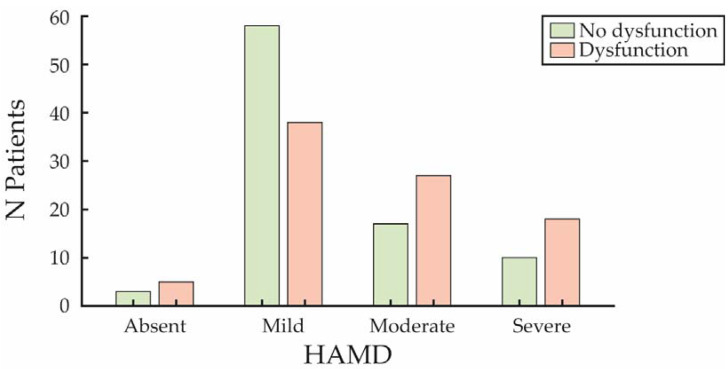
Sexual dysfunction by depression severity. The number of patients with (in red) and without (in green) sexual dysfunction across four depression categories.

In the mild depression category, 58 over 96 patients (about 60%) did not report SD. In contrast, SD became progressively more prevalent in patients with moderate (27 out of the total 44 patients, about 61%) and severe (18 out of 28 patients, about 64%) depression (Figure 4). Each depression category was analyzed separately to account for the heterogeneity of the sample distribution, and no direct comparisons were made between them.

## 4. Discussion

SD is a common issue in the general Italian population, with significant prevalence among older adults. Recent data indicate that SD affects approximately 38.2% men to 22.8% women [28], with prevalence rates increasing substantially with age. For instance, ED impacts 45.2% in men aged 40–70 years [29]. Similarly, SD is prevalent among women over 60 years old, with widely reported challenges, such as reduced libido, difficulty achieving orgasm, and pain during intercourse. The demand for assistance with SD has increased by 30% in recent years, reflecting a growing recognition of its impact on quality of life [29]. When compared to the general population, the prevalence of SD is significantly higher in individuals with PD. Indeed, in our study, 39% of females and 58% of males with PD reported SD. This finding suggests that, beyond age-related and general health factors, PD-specific mechanisms, such as dopaminergic pathway dysfunction, depression, and motor disability, may exacerbate SD. This comparison underscores the compounded burden of SD in PD patients, highlighting the critical need for tailored, multidisciplinary interventions to address this multifactorial and often-overlooked non-motor symptom. Previous studies have indicated that SD affects a significant proportion of individuals with PD [12,30].

The etiology of SD in PD patients is diverse, involving both motor and non-motor factors, including neurodegenerative and aging changes, polypharmacy side effects, and psychological aspects, such as depression and anxiety [6].

Additionally, hormonal imbalances play a significant role in PD-associated SD. Indeed, testosterone deficiency has been linked to reduced libido and ED in men [31], while changes in estrogen levels, particularly post-menopause, may exacerbate sexual health issues in women [18]. Prolactin dysregulation, often related to antidopaminergic treatments, can also negatively affect sexual desire and arousal [32]. These hormonal factors, combined with the neurodegenerative processes in PD, underscore the complexity of SD as well as the need for a holistic management approach [10]. Pharmacological treatments, such as antidepressants, and their effects on serotonergic pathways may also contribute to SD, further highlighting the multifactorial nature of this symptom [33].

In addition, mechanisms of neurodegeneration, such as genetic, inflammatory, and environmental factors, may also contribute to SD in PD patients. Genetic mutations, such as those in the LRRK2 and SNCA genes, have been associated with both motor and non-motor symptoms [10], including SD [10]. Chronic neuroinflammation and oxidative stress, key features of neurodegeneration, may further exacerbate autonomic dysfunction, impacting sexual health [32]. Environmental exposure to neurotoxins may also play a role by accelerating these processes. Vascular health, dopaminergic degeneration, and comorbidities, such as diabetes or hypertension, can further compound these issues [34].

In our previous work, we highlighted how gender difference, depression, type of relationship, and comorbidities can affect SD, emphasizing the importance of paying attention to sexual issues, also in women [14]. In this secondary analysis, the results further identify the key factors influencing SD, with gender, age, and depression emerging as significant predictors, as indicated by the GLME model. These findings, based on a refined cohort of PD patients, provide a more reliable evaluation of the factors influencing SD, underscoring the role of both biological and psychosocial contributors to SD in patients with PD.

A recent work by Souza et al. [35] found that motor disability and depression were significant predictors of SD in men with PD, while other factors, such as age, disease onset, and medication dosage, were not. Unlike our study, which included both genders, Souza et al. focused exclusively on male patients. Despite these differences, the findings of the role of depression in predicting long-term sexual health aligns with our results, which demonstrate that the severity of depression increases as the incidence of SD increases [35].

The significant effect of gender on SD observed in our study is consistent with the existing literature [12,31,34,36,37]. The research indicates that men generally report higher rates of SD compared to women, not only in PD but also in the general aging population and in those with other neurological disorders [12,36]. For instance, a meta-analysis found that men with PD were nearly twice as likely to experience SD compared to age-matched healthy controls, whereas no such association was found for women [34]. The greater stigma surrounding SD in men, coupled with societal expectations regarding sexual performance, may contribute to these differences [31]. Furthermore, physiological factors, such as more pronounced vascular contributions to ED in men, may account for the higher rates of SD in male patients [37].

In addition, the duration of disease may also play a critical role in SD, as patients with longer disease progression and greater motor impairment may experience worse NMS outcomes, including SD [38]. Although not directly addressed in this study, this aspect could be an important variable to consider in future analyses.

Age also emerged as a significant factor influencing SD in both men and women [10,31]. However, the steeper gradient observed in women suggests that aging may have a more pronounced effect on their SD. This could be related to hormonal changes, particularly those associated with menopause, which are known to significantly impact sexual health [39]. In contrast, men experience a more gradual decline in sexual function with age, likely due to slower hormonal changes [33]. Psychosocial factors, such as body image concerns and relationship dynamics, may further exacerbate the effects of aging on sexual health in women [31]. The interaction between these factors may explain the more significant age-related increases in SD observed among female patients. As age increases, SD is increasingly influenced by vascular factors, polypharmacy, and the degeneration of the mesolimbic pathway in PD patients, which plays a critical role in sexual desire [15].

Another aspect that warrants attention is the presence of comorbidities, such as diabetes and hypertension, which could influence SD outcomes in patients with PD [25,40]. Although these factors were not directly analyzed in the current study, they represent important clinical considerations in the management of SD and should be explored in future research.

The impact of depression on SD is particularly noteworthy. Our analysis shows that, as depression severity increases, the likelihood of SD also rises. This highlights the complex interplay between mental health and sexual function. Depression can induce physiological changes, such as altered neurotransmitter levels, as well as psychological effects, like low self-esteem and anxiety, all of which can contribute to SD [41]. Atlantis and Sullivan reported that individuals with depression have a 50–70% increased risk of developing SD, while those with SD have a 130–210% increased risk of experiencing depression [41]. The bidirectional relationship between SD and depression underscores the importance of addressing both conditions simultaneously.

**Figure 5:**
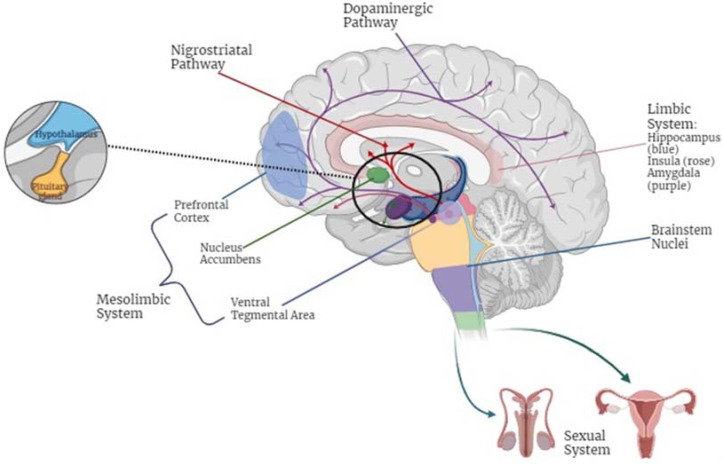
Neuroanatomical pathways involved in sexual dysfunction in Parkinson’s Disease patients. Legend: This figure highlights the key neural pathways and regions implicated in sexuality, many of which are disrupted in Parkinson’s Disease patients. The mesolimbic pathway (ventral tegmental area, nucleus accumbens, and prefrontal cortex) drives motivation, reward, and sexual desire. The prefrontal cortex (orbitofrontal and dorsolateral regions) oversees executive control and social judgment in sexual behavior. The limbic system (amygdala, hippocampus, and insula) manages emotional processing, memory, and bodily awareness essential for intimacy. The nigrostriatal pathway (substantia nigra and striatum) facilitates motor coordination and sensory–motor integration, often impaired in Parkinson’s Disease patients. The incertohypothalamic pathway (zona incerta and hypothalamus) regulates hormonal and autonomic responses, influencing erection and vaginal lubrication. The sensorimotor system ensures physical responsiveness and movement, while brainstem nuclei integrate autonomic and reflexive responses, such as an erection and orgasm. This integrative view underscores the interplay between these systems and their collective role in sexual function, particularly in the context of Parkinson’s Disease.

Dopaminergic pathways, which are often impaired in PD patients, play a critical role in sexual desire and performance [37]. Indeed, the important role of dopamine in the mesolimbic area has been demonstrated, as a mediator of reinforcement learning, implicated in the regulation of sexuality [42]. The neuroanatomy of SD in PD patients involves a complex interplay of brain regions and pathways responsible for both motor and non-motor functions (see Figure 5).

Key areas include (i) the hypothalamus, which regulates hormonal release and autonomic functions; (ii) the prefrontal cortex, associated with emotional regulation and decision making; and (iii) the basal ganglia, which play a central role in dopaminergic signaling [10]. Specifically, among the dopaminergic pathways involved in SD, we should take into account (i) the mesolimbic dopaminergic system, which plays a role in the anticipatory/motivational phase; (ii) the nigrostriatal dopaminergic system, which provides sensory–motor coordination, for consummation; and (iii) incertohypothalamic dopaminergic neurons, involved in sexual motivation through mesolimbic dopaminergic neuron interactions [32,43,44,45,46]. Damage to these regions contributes to impairments in sexual desire, arousal, and performance [32]. Additionally, brainstem nuclei, such as the locus coeruleus and the dorsal motor nucleus of the vagus, are implicated in autonomic regulation, further exacerbating SD in PD patients [7]. These disruptions are compounded by peripheral mechanisms, including autonomic neuropathy and impaired feedback between central and peripheral systems.

Iatrogenic SD in PD patients may present as either hypersexuality, due to a direct effect of dopaminergic stimulation induced by PD drugs (as dopamine is known to enhance libido and sexual arousal [47]) or hyposexuality, due to the serotoninergic effect of many antidepressants (as serotonin has a clear inhibitory effect on sexuality). Indeed, antidepressants with a predominant serotoninergic action may lead to SD, particularly in the form of anorgasmia and ejaculatory disorders [48]. These factors may have important clinical implications, particularly in managing sexual health in patients with depression and aging-related concerns. Tailored interventions that consider gender-specific and age-related factors could help mitigate the impact of aging and depression on SD. For instance, targeted therapies for women experiencing menopause or for men in later life stages may improve sexual health outcomes [39]. Additionally, integrating sexual health into the treatment of depression could provide a more holistic approach to care, ultimately enhancing the quality of life for these patients. It would also be beneficial to assess the interaction between the key factors identified in this study, such as the interaction between age and gender or depression and gender, to gain a more nuanced understanding of their combined effects on SD. This approach could offer deeper insights into how demographic and psychological factors interact to shape sexual health outcomes in PD patients. The multifactorial nature of SD in PD patients highlights the need for comprehensive and personalized treatment approaches. Healthcare professionals should proactively assess sexual health as part of their routine care for patients with PD, utilizing tools that capture the full spectrum of SD and its impact on patients and their partners.

Future research should focus on developing interventions for sexual dysfunction (SD) that consider individual patient needs, including demographic characteristics, disease stage, and comorbidities [48].

This secondary analysis has several limitations due to the study design. As a multicenter cross-sectional study, the data represent a snapshot in time, limiting the ability to establish causal relationships between the variables considered. The heterogeneity among participating centers may have introduced variations in clinical practices and data collection protocols, affecting the comparability of the results. Moreover, the cross-sectional nature of the study prevents the establishment of causal relationships, as longitudinal data would be required to explore how these factors evolve over time. Although the GLME model was employed to account for individual variability, the binomial distribution of the dysfunction variable might limit the model’s sensitivity to more subtle differences. Furthermore, the presence of missing or incomplete data may reduce internal validity and statistical power, potentially compromising the generalizability of the findings to the broader population of patients with PD.

Another limitation of our study is that we did not stratify patients based on PD subtypes (e.g., tremor-dominant versus akinetic-rigid forms), which may influence the relationship between depression and SD. Future research should explore the potential interaction between PD subtypes, depression, and SD to provide a more comprehensive understanding of these complex relationships. Additionally, longitudinal studies may be useful to better capture the progression of dysfunction over time and to clarify causal relationships between the identified factors. Finally, efforts to incorporate stratification techniques based on the subtypes of PD and disability severity could underline the relationship between PD progression and SD.

## 5. Conclusions

This study demonstrates that age, gender, and depression are important predictors of SD in patients with PD. Although SD is a common NMS in PD patients, causing distress for both patients and partners, it is often neglected in clinical practice and the research on this important issue remains limited. A multidisciplinary approach, including nursing and coaching, is therefore essential to improve sexual health and quality of life for these patients. Clinicians should be aware of this important issue and consider SD when managing PD, as disregarding sexuality in this patient population is no longer acceptable, given the growing recognition of sexual health as the one of the most significant indicators of quality of life.

## Figures and Tables

**Table 1:** Demographic and clinical characteristics of the sample. Continuous variables are expressed as mean ± standard deviation; categorical variables are expressed as frequencies (%). Significant values are denoted as ** *p* < 0.01. Legend: Fisher (Fisher’s exact test); χ^2^ (chi-squared test); HAMD (Hamilton Rating Scale for Depression); SD (sexual dysfunction); U (Mann–Whitney U test).

Characteristic	All	Female	Male	*p*-Value	Test Type
Participants	176	75 (42.61)	101 (57.39)		
Age	67.97 ± 8.31	67.13 ± 8.26	68.59 ± 8.33	0.16	U
Education				0.43	χ^2^
Elementary school	62 (35.23)	31 (41.33)	31 (30.69)		
Middle school	46 (26.14)	18 (24)	28 (27.72)		
High school	35 (19.89)	15 (20)	20 (19.8)		
University degree	33 (18.75)	11 (14.67)	22 (21.78)		
Presence of son/daughter	169 (96.02)	70 (93.33)	99 (98.02)	0.14	Fisher
HAMD				0.68	χ^2^
Absent	8 (4.55)	4 (5.33)	4 (3.96)		
Mild	96 (54.55)	41 (54.67)	55 (54.46)		
Moderate	44 (25)	16 (21.33)	28 (27.72)		
Severe	28 (15.91)	14 (18.67)	14 (13.86)		
SD	88 (50)	29 (38.67)	59 (58.42)	0.01 **	χ^2^

## Data Availability

Data will be available upon request from the corresponding authors.
